# Drug-coated balloon versus drug-eluting stent in acute myocardial infarction: A protocol for systematic review and meta-analysis

**DOI:** 10.1097/MD.0000000000027259

**Published:** 2021-11-05

**Authors:** Baoyu Geng, Zhe Liu, Guangzhi Feng, Jun Jiang

**Affiliations:** a Department of Cardiology, Taixing People's Hospital, Jiangsu, China; b Department of Cardiology, The Second Affiliated Hospital of Zhejiang University School of Medicine, Zhejiang, China.

**Keywords:** acute myocardial infarction, drug-coated balloon, drug-eluting stents, meta-analysis, protocol

## Abstract

**Background:**

Previous studies comparing the published literature on drug-eluting stents (DES) and drug-coated balloon (DCB) have drawn divergent conclusions, as these studies are limited by small sample sizes. To overcome these limitations, we thus conducted a high-quality systematic review and meta-analysis to assess the efficacy and safety of DCB versus DES for patients with acute myocardial infarction (AMI). It was hypothesized that DCB use at the AMI is associated with decreased risk of cardiovascular disease and death.

**Methods:**

The electronic databases Embase, Medline, PubMed, and Cinahl were searched from the earliest available date until August 2021. Study included in our study had to meet all of the following inclusion criteria: all randomized controlled trials to assess the efficacy and safety of DES versus DCB in the treatment of AMI were considered eligible for analysis; participants received DCB or DES; reporting the available data on cardiac death, all-cause death, myocardial infarction, target lesion revascularization, target vessel revascularization, major adverse cardiac events, and stent thrombosis. Review Manager Software (v 5.3; Cochrane Collaboration) was used for the meta-analysis. Two of us independently assessed the risk of bias in the included studies using parameters defined in the Cochrane Handbook for Systematic Reviews of Interventions criteria.

**Results:**

It was hypothesized that DCB use at the AMI is associated with decreased risk of cardiovascular disease and death.

**Registration number:**

10.17605/OSF.IO/AVTYW.

## 1 Introduction

Cardiovascular disease is a leading cause of mortality and morbidity worldwide, with an estimated 8.14 million people worldwide died from acute myocardial infarction (AMI) in 2013.^[1]^ Primary percutaneous coronary intervention with drug-eluting stents (DES) has become one of the most commonly used methods for the treatment of ST-elevation myocardial infarction. The introduction of bare metal stents significantly reduced revascularization rates after primary percutaneous coronary intervention compared to standard balloon angioplasty alone. With the development of DES, these ratios fell even further.^[2,3]^ However, routine stent placement did not mean a reduced rate of cardiac death or recurrent myocardial infarction. In addition, permanent vascular implants are associated with an increased risk of stent thrombosis, which impair the vasomotor function of the culprit coronary arteries.^[4]^

Considering the lack of superiority in terms of hard clinical endpoints and the potential short- and long-term drawbacks of stent implantation, angioplasty with drug-coated balloon (DCB) without stents is a good strategy for the treatment of AMI.^[5]^ The advantage of DCB is that it provides a uniform drug distribution at a short sustained exposure and high topical drug dose, thus preventing the disadvantages of conventional old balloon angioplasty.^[6]^ In addition, the need for stent implantation can avoid long-term drawbacks such as stent thrombosis and coronary vasomotor responses or vascular geometry disturbances. Finally, this strategy may reduce the long-term need for dual antiplatelet therapy.^[7,8]^

Several recent studies have showed that both DES and DCB outperform other AMI intervention strategies.^[9,10]^ In addition, the recent European Society of Cardiology guidelines for myocardial revascularization also recommend DCB and DES for patients with AMI.^[11]^ However, previous randomized controlled studies^[6,12]^ and meta-analysis^[13]^ comparing the published literature on DES and DCB have drawn divergent conclusions, as these studies are limited by small sample sizes. In recent years, several large randomized controlled trials have been published. To overcome these limitations, we thus conducted a high-quality systematic review and meta-analysis to assess the efficacy and safety of DCB versus DES for patients with AMI. It was hypothesized that DCB use at the AMI is associated with decreased risk of cardiovascular disease and death.

## 2 Materials and methods

### 2.1 Data sources and search strategy

The systematic literature review was structured to adhere to Preferred Reporting Items for Systematic Reviews and Meta-analyses guidelines, which included requirements deemed essential for the transparent reporting of results. The electronic databases Embase, Medline, PubMed, and Cinahl were searched from the earliest available date until August 2021. The concepts of population, intervention, control, outcome, and design were combined with the “AND” operator. The population was defined as participants with AMI. The intervention was defined as a participant receiving DCB. The control group was defined as participants receiving DES. The outcomes were cardiac death, all-cause death, myocardial infarction, target lesion revascularization, target vessel revascularization, major adverse cardiac events, and stent thrombosis. The design was a randomized controlled trial. All articles were imported into the bibliographic software and screened for duplicates. Two reviewers independently screened the title and abstract of each article using predetermined eligibility criteria. Reference lists of included articles were hand-searched and citation tracking applied using Google Scholar to identify any further articles for inclusion. The review protocol has been registered on Open Science Framework registries. Ethical approval and patient consent were not required because this study was a literature-based study.

### 2.2 Eligibility criteria

Study included in our study had to meet all of the following inclusion criteria: all randomized controlled trials to assess the efficacy and safety of DES versus DCB in the treatment of AMI were considered eligible for analysis; participants received DCB or DES; reporting the available data on cardiac death, all-cause death, myocardial infarction, target lesion revascularization, target vessel revascularization, major adverse cardiac events, and stent thrombosis. Studies with overlapping data or insufficient data to calculate or extract effect estimates would be excluded. Case reports, biochemical trials, letters, and reviews would also be eliminated.

### 2.3 Data extraction

The data was extracted in duplicate. The reasons of exclusion at this stage were summarized. Results were recorded on trial data extraction forms and Excel spreadsheets. Data extracted related to: country and study date; participants (indication, age, sex); inclusion and exclusion criteria; intervention content and control group; setting, timing, duration and intensity of the intervention; follow-up time; subsequent losses and their causes; and the outcomes. For the results reported as continuous variables, the mean and standard deviation were extracted. If the results were reported as mean and confidence intervals, or median and quartile spacing, the appropriate conversion would be applied. If necessary, the lead author of the study would be contacted for missing data. We also asked whether any results not reported in their publications had been collected. If the author had provided information to other reviewers, the data would be included in our analysis and acknowledged appropriately.

### 2.4 Statistical analysis

Review Manager Software (v 5.3; Cochrane Collaboration) was used for the meta-analysis. Extracted data were entered into Review Manager by the first independent author and checked by the second independent author. Risk ratio with a 95% confidence interval or standardized mean difference with 95% CI were assessed for dichotomous outcomes or continuous outcomes, respectively. The heterogeneity was assessed by using the Q test and I^2^ statistic. An I^2^ value of <25% was chosen to represent low heterogeneity and an I^2^ value of >75% to indicate high heterogeneity. All outcomes were pooled on random-effect model. A *P* value of <.05 was considered to be statistically significant.

### 2.5 Study quality assessment

Two of us independently assessed the risk of bias in the included studies using parameters defined in the Cochrane Handbook for Systematic Reviews of Interventions criteria. Differences were resolved through discussion and consensus among reviewers. Based on the information provided by the included studies, each item was recorded as high risk of bias, low risk of bias, or unclear. Two reviewers independently assessed the quality of the body of evidence for different outcomes using the grades of recommendation, assessment, development, and evaluation approach, a proven and widely practiced tool for assessing the quality of scientific evidence. Based on the grades of recommendation, assessment, development, and evaluation approach, we assessed 5 areas, ranking the strength of evidence for each result.

## 3 Discussion

Several recent studies have showed that both DES and DCB outperform other AMI intervention strategies.^[9,10]^ In addition, the recent European Society of Cardiology guidelines for myocardial revascularization also recommend DCB and DES for patients with AMI.^[11]^ However, previous randomized controlled studies^[6,12]^ and meta-analysis^[13]^ comparing the published literature on DES and DCB have drawn divergent conclusions, as these studies are limited by small sample sizes. In recent years, several large randomized controlled trials have been published. To overcome these limitations, we thus conducted a high-quality systematic review and meta-analysis to assess the efficacy and safety of DCB versus DES for patients with AMI. It was hypothesized that DCB use at the AMI is associated with decreased risk of cardiovascular disease and death.

## Author contributions

**Conceptualization:** Baoyu Geng, Zhe Liu.

**Data curation:** Baoyu Geng, Zhe Liu.

**Formal analysis:** Baoyu Geng, Zhe Liu.

**Funding acquisition:** Jun Jiang.

**Investigation:** Baoyu Geng, Zhe Liu.

**Methodology:** Guangzhi Feng.

**Resources:** Jun Jiang.

**Software:** Guangzhi Feng.

**Supervision:** Jun Jiang.

**Validation:** Guangzhi Feng.

**Visualization:** Guangzhi Feng.

**Writing - original draft:** Baoyu Geng.

**Writing - review & editing:** Jun Jiang.
